# The Perioperative Surgical Home: how can it make the case so everyone wins?

**DOI:** 10.1186/1471-2253-13-6

**Published:** 2013-03-14

**Authors:** Thomas R Vetter, Lee A Goeddel, Arthur M Boudreaux, Thomas R Hunt, Keith A Jones, Jean-Francois Pittet

**Affiliations:** 1 Department of Anesthesiology, University of Alabama School of Medicine, JT862, 619 19th Street South, Birmingham, AL, 35249-6810, USA; 2 Department of Anesthesiology, University of Alabama School of Medicine, 619 19th Street South, JT-920, Birmingham, AL, 35249-6810, USA; 3 Department of Anesthesiology, University of Alabama School of Medicine, 619 19th Street South, JT-823, Birmingham, AL, 35249-6810, USA; 4 Division of Orthopedics, University of Alabama School of Medicine, 1313 13th Street South, OSB Suite 201, Birmingham, AL, 35205, USA; 5 Department of Surgery, University of Alabama School of Medicine, 1313 13th Street South, OSB Suite 201, Birmingham, AL, 35205, USA; 6 Department of Anesthesiology, University of Alabama School of Medicine, 619 19th Street South, JT-804, Birmingham, AL, 35249-6810, USA; 7 Department of Anesthesiology, University of Alabama School of Medicine, 619 19th Street South, JT-926, Birmingham, AL, 35249-6810, USA

**Keywords:** Surgical home, Perioperative care, Healthcare outcomes, Comparative effectiveness, Healthcare economics, Patient satisfaction, Patient-centered care

## Abstract

**Background:**

Varied and fragmented care plans undertaken by different practitioners currently expose surgical patients to lapses in expected care, increase the chance for operational mistakes and accidents, and often result in unnecessary care. The Perioperative Surgical Home has thus been proposed by the American Society of Anesthesiologists and other stakeholders as an innovative, patient-centered, surgical continuity of care model that incorporates shared decision making. Topics central to the debate about an anesthesiology-based Perioperative Surgical Home include: holding the gains made in anesthesia-related patient safety; impacting surgical morbidity and mortality, including failure-to-rescue; achieving healthcare outcome metrics; assimilating comparative effectiveness research into the model; establishing necessary audit and data collection; a comparison with the hospitalist model of perioperative care; the perspective of the surgeon; the benefits of the Perioperative Surgical Home to the specialty of anesthesiology; and its associated healthcare economic advantages.

**Discussion:**

Improving surgical morbidity and mortality mandates a more comprehensive and integrated approach to the management of surgical patients. In their expanded capacity as the surgical patient’s “perioperativist,” anesthesiologists can play a key role in compliance with broader set of process measures, thus becoming a more vital and valuable provider from the patient, administrator, and payer perspective. The robust perioperative databases created within the Perioperative Surgical Home present new opportunities for health services and population-level research. The Perioperative Surgical Home is not intended to replace the surgeon’s patient care responsibility, but rather leverage the abilities of the entire perioperative care team in the service of the patient. To achieve this goal, it will be necessary to expand the core knowledge, skills, and experience of anesthesiologists. Anesthesiologists will need to view becoming perioperative physicians as an expansion of the specialty, rather than an abdication of their traditional intraoperative role. The Perioperative Surgical Home will need to create strategic added value for a health system and payers. This added value will strengthen the position of anesthesiologists as they navigate and negotiate in the face of finite, if not decreasing fiscal resources.

**Summary:**

Broadening the anesthesiologist’s scope of practice via the Perioperative Surgical Home may promote standardization and improve clinical outcomes and decrease resource utilization by providing greater patient-centered continuity of care throughout the preoperative, intraoperative, and postoperative periods.

## Background

Varied and fragmented care plans undertaken by different practitioners currently expose surgical patients to lapses in expected care, increase the chance for operational mistakes and accidents, and result in unnecessary care [1-3]. Standardization of perioperative processes has become increasingly recognized as needed to optimize resource utilization, quality, and patient safety [4,5]. Likewise, the medical community and the public are increasingly embracing shared decision making, a process by which healthcare choices are made jointly by the practitioner and the patient [6,7]. Akin to the Medical Home model that has been developed in the primary care practice setting [8-10], the Perioperative Surgical Home has thus been proposed by the American Society of Anesthesiologists and other stakeholders as an innovative, patient-centered, surgical continuity of care model that incorporates shared decision making [11,12]. This broadening the anesthesiologist’s scope of practice to provide continuity of care throughout the preoperative, intraoperative, and postoperative periods may promote such standardization and shared decision making, thus improving clinical outcomes and decreasing unnecessary resource utilization [3,13].

In this paper, we present the PSH model we have developed at the University of Alabama at Birmingham (UAB) as a prototypic example. We then discuss a series of topics central to the debate about the implementation of such an anesthesiology-based PSH, specifically: 1) holding the gains made in anesthesia-related patient safety, 2) impacting surgical morbidity and mortality, including inpatient “failure to rescue,” 3) achieving healthcare outcome metrics, 4) assimilating comparative effectiveness research into the model, 5) establishing audit and data collection, 6) a comparison with the hospitalist model of perioperative care, 7) the perspective of the surgeon, 8) the benefits of the PSH to the specialty of anesthesiology, and 9) its associated healthcare economic advantages.

### The UAB Perioperative Surgical Home Model

**Figure 1:**
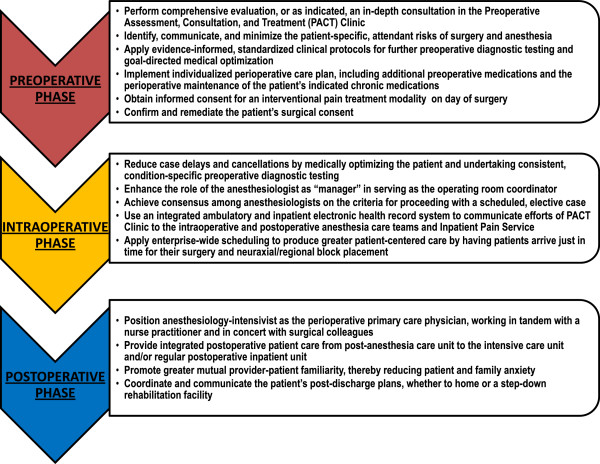
Integration of the preoperative, intraoperative, and postoperative phases of patient care with the Perioperative surgical home model.

The UAB PSH model seeks to integrate the three well-recognized but frequently fragmented preoperative, intraoperative, and postoperative phases of patient care (Figure 1). It is fundamentally based on the anesthesiologist serving as the surgical patient’s primary “perioperativist,” who provides a seamless continuity of current best practices of care, while actively engaging the patient, family, and other health care providers.

#### Preoperative phase

Once the mutual decision has been by the patient and surgeon to proceed to surgery, the patient is referred to the UAB Highlands Preoperative Assessment, Consultation and Treatment (PACT) Clinic. During their initial PACT Clinic visit, patients are evaluated by a Certified Registered Nurse Practitioner (CRNP). A group of these mid-level providers works together with an attending anesthesiologist with a strong self-identified interest in preoperative management, who is assigned daily to the PACT Clinic.

While a pre-anesthetic patient assessment has been a longstanding required element of any anesthetic, it has been historically performed in close proximity to the scheduled surgery and has routinely only collected a limited set of clinical data. In patients with a greater chronic disease burden, such a perfunctory pre-anesthetic assessment does not permit the more comprehensive evaluation, or as indicated, the consultation, which our PACT Clinic affords.

The goals of the PACT Clinic are to identify, to communicate, and whenever possible to minimize the patient-specific, attendant risks of surgery and anesthesia. This process relies heavily on evidence-based, standardized clinical protocols for further preoperative diagnostic testing and treatment. This medical optimization includes not only beta-blocker, statin, and anticoagulant therapy but also the administration of subcutaneous recombinant erythropoietin and intravenous iron for preoperative anemia to reduce or eliminate surgical allogeneic blood transfusions.

Patient education, engagement, and empowerment are emphasized by the PACT Clinic. As promulgated by the Enhanced Recovery After Surgery (ERAS) Society [14,15], detailed information is given to patients before their surgery and anesthesia to diminish anxiety and enhance postoperative recovery and hasten hospital discharge. Preoperative psychological intervention, aimed at decreasing patient anxiety, is undertaken to improve wound healing and recovery from surgery. This includes personal counseling, pamphlets and multimedia information containing explanations of the planned procedure, along with tasks that the patient is encouraged to undertake, to improve postoperative feeding, early postoperative mobilization, pain control, and respiratory physiotherapy—thus reducing the incidence of complications [14,15].

Controlling postoperative pain is also paramount. Patients are thus consistently educated and empowered by the PACT Clinic staff about their right to effective pain management and options. An individualized pain management plan is developed, including preoperative oral medications and the assured perioperative maintenance of the patient’s chronic opioids. Achieving preventive analgesia and reducing chronic post-surgical pain is also emphasized. This plan is directly communicated to the intraoperative anesthesia care team and our well-established Inpatient Pain Service. When indicated, the PACT Clinic obtains a separate written informed consent for an interventional pain treatment modality, lessening the patient’s stress of making such a decision on the day of surgery. This also increases efficiency on the day of surgery, reducing stress among providers. The PACT Clinic also confirms the accuracy and completeness of the patient’s written surgical consent and prompts the surgeon to remedy any deficiencies before the day of surgery.

#### Intraoperative phase

Case delays and cancellations on the day of surgery waste resources and are frustrating for all involved, but in particular, for the patient and family. Our PSH model mitigates the risk of such case delays and cancellations not only by medically optimizing the patient but also by achieving strong consensus among our group of anesthesiologists as to the criteria for proceeding with a scheduled, elective case. Our fully integrated electronic medical record assures that the product of the comprehensive efforts of the PACT Clinic is communicated to the intraoperative anesthesia care team and the Inpatient Pain Service, thus enabling them to deliver optimal, individualized patient care. These care providers can further reassure the patient and family by way of their clear awareness of the patient’s unique circumstances. While our PSH model does not stipulate the choice of anesthetic technique or specific medication to be used, our departmental Section on Quality and Patient Safety and Section on Information Services have developed an innovative anesthesiology dashboard. This real-time electronic dashboard allows anesthesia providers to access the daily annotated surgical schedule, to review current departmental patient care protocols, and to track and compare their individual performance in meeting established quality and safety benchmarks.

#### Postoperative phase

Given the ever-present demand for maximum intraoperative productivity and efficient resource use, immediate postoperative care is understandably yet typically only addressed by the surgical attending very early in the morning and late in the day. In an academic medical center, most such patient care may also be delegated to more junior surgical team members. Inadequate postoperative communication and hand-offs among health care providers are a common root cause of patient complications. By contrast, in our PSH model, a single anesthesia-intensivist attending works in tandem with the same group of mid-level providers (CRNPs) and a registered nurse (RN) case coordinator to consistently provide more focused and integrated postoperative patient care—from the post-anesthesia care unit (PACU) to the intensive care unit and/or the regular inpatient unit. This postoperative care team interacts closely with our anesthesiology-based Inpatient Pain Service to assure optimal pain assessment and treatment—including the timely transition to oral analgesics and adjuvant medications. The resulting robust continuity of care enhances the patient’s postoperative experience, in part by promoting greater mutual familiarity and thereby reducing patient and family anxiety. As the hospital discharge approaches, this anesthesiology postoperative team coordinates and communicates the patient’s post-discharge plans, whether to home or a step-down rehabilitation setting, to the patient’s primary care physician. This seamless transition from the inpatient to the outpatient setting will be very important to realizing the full benefits of continuity of care. Ideally, the patient’s *Perioperative Surgical Home* team interfaces with patient’s *Medical Home* team, thereby promoting “shared care” and optimizing patient compliance and outcomes and reducing post-discharge emergency department visits and hospital readmissions—including for inadequate postoperative analgesia.

## Discussion

### Holding the gains in anesthesia-related patient safety

The Institute of Medicine (IOM) has recognized the specialty of anesthesiology for progressively decreasing mortality rates in the United States (US), from 1 death per 1,000 anesthetics in 1940 to contemporary estimates of 1 death per 15,000 anesthetics [16-18]. This improved safety has notably occurred despite an ageing population and escalating patient disease burden in the US. The number of Americans age 65 or older increased from 9 million in 1940 to 40 million in 2010. This figure is projected to reach 55 million by 2020 and 72 million by 2030 [19]. The prevalence of chronic diseases has concomitantly increased [20]. By 2020, 157 million US citizens are predicted to have one chronic disease, and 81 million will have multiple such conditions [21,22]. Both increased age and prevalence of chronic diseases have been independently associated with increased surgical mortality [23,24]. Despite this increased associated risk, the rate of surgical procedures in the elderly population has particularly increased [25,26]. Maintaining or improving on 20^th^ century gains in anesthesia-related mortality will thus be challenging. As a specialty, anesthesiology must remain firmly committed and continue to work diligently on established tenets as well as new patient safety principles and concepts [27]. It will also require that health care providers, administrators, and policy makers in the United States collectively implement a more comprehensive and integrated approach to the management of patients undergoing surgery. The PSH model is a novel yet promising such integrated management approach.

### Impacting surgical morbidity and mortality

Surgical death rates reportedly vary widely across hospitals in the United States, from 3.5% in very-low-mortality hospitals to 6.9% in very-high-mortality hospitals [28]. Payers (e.g., Centers for Medicare and Medicaid Services) and regulators (e.g., The Joint Commission) are currently focusing on ways of reducing postoperative complications, which may be one approach to reducing this observed variability in surgical mortality. However, based upon data from the American College of Surgeons National Surgical Quality Improvement Program (NSQIP), from 2005 through 2007, hospitals with either very high mortality or very low mortality reported similar rates of overall complications and of major complications. In contrast, mortality in patients with major NSQIP-defined complications (see section List of Major Surgical Complications) [29] was significantly greater in hospitals with very high overall mortality compared with those with very low overall mortality (21.4% versus 12.5%) [28]. Therefore, in addition to efforts aimed primarily at *avoiding* complications, reducing mortality associated with inpatient surgery will likely require greater attention to the *timely recognition and management* of complications once they occur [28].

Major Surgical Complications from the American College of Surgeons National Surgical Quality Improvement Program (NSQIP) [29]:

•Mortality

•Pneumonia

•Unplanned intubation

•Prolonged mechanical ventilation (> 48 hours)

•Deep venous thrombosis

•Pulmonary embolism

•Deep wound infection

•Organ-space infection

•Acute renal failure

•Myocardial infarction

•Stroke

•Urinary tract infection

•Septic shock

•Postoperative bleeding requiring transfusion

•Vascular graft loss

•Fascial dehiscence

Based upon Medicare beneficiary data from 2005 to 2006, complication rates in patients undergoing six major inpatient surgical procedures were also similar at the worst and best hospitals (36.4% versus 32.7%), but the worst hospitals had mortality rates 2.5-fold higher than the best hospitals [30]. Furthermore, “failure to rescue” rates were much higher at the worst compared with the best hospitals (16.7% vs. 6.8%) [30]. Based upon a growing body of literature, there is a growing consensus that variations in surgical mortality are due in part to such “failure to rescue” (mortality among patients with complications) rather than differences in postsurgical complications [31].

Failure-to-rescue (FTR) was first defined in 1992 by Silber and colleagues as hospital deaths after adverse occurrences such as postsurgical complications [32,33], and in 2001 it was identified by the IOM as one of the key areas for improvement in patient safety [33,34]. Contributors to FTR have been broadly categorized as the lack of a *timely* response (prompt recognition of the complication) and an *appropriate* response (correct management and treatment) [28,33]. An abundance of retrospective data supports that adverse events in general ward (non-ICU) patients are preceded by a significant period—on the order of hours—of physiologic deterioration [33]. Thus, the lack of early recognition and treatment of physiologic decline plays a major role in the inpatient FTR problem, including in postoperative patients [33]. Given not only the rigorous continuity of care afforded by this new care model, but also the innate heightened awareness and expertise among its participating anesthesia perioperativists, the PSH should allow for a timelier and more appropriate response to patient physiologic derangement, thus reducing FTR event rates, major NSQIP-defined complications (see section List of Major Surgical Complications), and associated surgical morbidity and mortality.

### Achieving key healthcare metrics with the perioperative surgical home model

The Patient Protection and Affordable Care Act (PPACA) of 2010 aims to control cost by transforming healthcare delivery through comparative effectiveness research, new infrastructure models to deliver more cost effective and coordinated care, and incentives based upon changes in reimbursement [35,36]. In this new healthcare paradigm, providers—including anesthesiologists—will be paid not just for the quantity of services they provide but for how well they deliver those services according to standardized metrics [37,38].

The central elements of current and likely any future US health reform will link payment to quality via “pay-for-performance” and “value-based purchasing” models and mandate linked reporting initiatives [39]. As set forth by the Centers for Medicare and Medicaid Services (CMS), these quality metrics include appropriate care measures, hospital-acquired conditions, and patient-reported scores on the Hospital Consumer Assessment of Healthcare Providers and Systems Survey (HCAHPS) [40]. In the near future, the initial CMS clinical process and patient experience measures will expand in number and financial (payment) ramifications [41].

The intraoperative anesthesiologist can only minimally impact a hospital’s performance on healthcare metrics (e.g., antibiotic administration, temperature control). However, in their expanded capacity as the surgical patient’s primary *perioperativist*, anesthesiologists can play a key role in achieving compliance with broader process measures, like those of the Surgical Care Improvement Project (SCIP) (see Section List of Performance Metrics) [42]—ultimately improving patient outcomes. By doing so, the anesthesiologist will become a more vital and valuable provider from the patient, administrator, and payer perspective.

Surgical Care Improvement Project (SCIP) Performance Metrics [42]:

•Beta Blocker during the Perioperative Period

•Prophylactic Antibiotic within 1 hour of incision

•Prophylactic Antibiotic Selection for Surgical Patients

•Prophylactic Antibiotic Discontinued within 24 hours

•Cardiac Surgery Patients with Controlled 6 A.M. Postoperative Blood Glucose

•Surgery Patients with Appropriate Hair Removal

•Urinary Catheter Removal within Two Days of Surgery

•Perioperative Temperature Management

•VTE Prophylaxis Ordered prior to Surgery

•VTE Prophylaxis Received within 24 Hours of Surgery

VTE: venous thromboembolism.

### Assimilating comparative effectiveness research into the perioperative surgical home model

The American Recovery and Reinvestment Act (ARRA) of 2009 charged the Institute of Medicine (IOM) with establishing a national comparative effectiveness research (CER) agenda [43,44]. The IOM has defined CER as the “the generation and synthesis of evidence that compares the benefits and harms of alternative methods to prevent, diagnose, treat, and monitor a clinical condition or to improve the delivery of care” [45]. “Health Care Delivery Systems” was the highest ranked IOM CER priority [45,46]. The new PSH model falls squarely in this highest ranked CER category.

Nearly every other developed country that has successfully reformed its healthcare delivery system has instituted some form of CER to prevent proposed initiatives from inadvertently harming patients and to foster intelligent changes that improve both the efficiency and the quality of care [47,48]. However, currently, nationwide capacity for healthcare performance data collection is inadequate to meet the goals of healthcare reform in the United States [49]. The PSH also establishes continuity of perioperative data collection, whereby multiple providers document patient information in standardized formats in a single electronic health record. This should create more consistent, complete, and externally valid databases—one of the tenets of CER [44]. Including patients who are more typical of those seen in day-to-day practice will more effectively answer the practicing clinician’s question of “do the results apply to patients in my practice?” [43,50]. Beyond generating mandated healthcare performance metrics, the robust perioperative databases created within a PSH model present a new opportunity for perioperative health services and population-level research. The comparative effectiveness and cost-efficiency of an anesthesiology-based PSH model must be demonstrated. Just as with the patient-centered Medical Home [10,51-53], a measurement and research agenda thus needs to be created for the patient-centered Perioperative Surgical Home—something we have done with our prototypic model at UAB.

### Establishing audit and data collection

Establishing audit and data collection is fundamental to the goals and objectives of the PSH, yet the required medical informatics is perhaps the greatest hurdle to be overcome in many hospitals. Although most anesthesiology departments and groups have quality improvement committees that are responsible for tracking complications, performing case review, and holding morbidity and mortality conferences, most are not organized to handle the implementation of major practice change. Therefore, in an effort to improve the quality and safety of the care we deliver, in 2007 the UAB Department of Anesthesiology developed a Section on Quality and Patient Safety [54], with the dedicated organization and resources needed for sustained success (“holding the gains”) [55-57]. Our hospital-level quality assurance, performance improvement, and patient safety programs are requisite in the United States for (a) demonstrating CMS Hospital Value-Based Purchasing Program payment criteria [58], (b) maintaining accreditation by The Joint Commission and CMS conditions of participation, and (c) achieving meaningful use of electronic health records set forth by CMS EHR Incentive Programs [59]. While our departmental Section on Quality and Patient Safety is located in an academic medical center, its interface and data sharing with corresponding hospital medical informatics infrastructure are feasible and applicable in community hospitals. This hospital medical informatics infrastructure will readily assist us in audit and data collection for assessing the effects of our PSH model on current and future key healthcare metrics described earlier.

### A comparison with the hospitalist model of perioperative surgical care

Although the potential full scope and role of a perioperative primary care physician has yet to be provided by a single specialty, internal medicine hospitalists and anesthesiologists have been most involved in the perioperative care of surgical patients [60,61]. Interestingly, there has been an attempt at a few institutions (e.g., UC San Francisco and Vancouver General Hospital) to develop a surgical hospitalist model, staffed by general surgeons, to provide timely and high-quality emergency surgical care and to enhance patient and referring provider satisfaction [62-64]. While this surgical hospitalist model may be a viable solution to diminishing access to emergency surgical care in the US and Canada, it will likely not solve the nationwide need for more comprehensive medical management of increasingly complex surgical patients.

The medical co-management of complex, high-risk surgical patients by perioperative physicians has become a common practice, particularly for orthopedic, cardiothoracic, and neurosurgical services [65]. In North America, most of this medical co-management of surgical patients is currently provided by internal medicine hospitalists. The co-management of surgical patients by internal medicine hospitalists has been prospectively evaluated by only few studies, which have yielded variable results [66]. Three studies have shown that hospitalist care leads to lower mortality, shorter hospital length of stay, and/or lower total inpatient costs [67-69]. However, two other studies, involving orthopedic and neurosurgical patients, did not demonstrate a significant effect on patient outcomes, satisfaction, or cost [70,71]. These divergent findings may support the common-sense notion that hospitalists most benefit patients who are sick, frail, and medically or socially complex [72].

To further develop and expand the role of internal medicine hospitalists in perioperative care is associated with two major problems. First, during their residency, internal medicine hospitalists do not have specific training or adequate exposure in managing complex surgical patients [72,73]. Second, there has been a decreasing interest among medical students in primary care careers, coinciding with increasing indebtedness for medical trainees, the ever-widening gap in salaries between primary care and specialist physicians, an exponential increase in primary care functions, and worsening burnout among practicing physicians who are expected to deliver more services in less time [9,74]. The resulting looming shortage of internists, particularly in primary outpatient care, raises the question whether we can afford to expand their breadth of practice, which could further exacerbate this workforce shortage [65]. Nevertheless, it must be demonstrated that anesthesiologists can provide greater “value” than internal medicine hospitalists.

### The perspective of the surgeon

The Surgical Home is not intended to replace the surgeon’s patient care responsibility, but rather as a way to leverage the talents and abilities of the entire perioperative care team in the service of the patient. From a surgeon’s perspective, the Surgical Home model can create value in three primary ways. First it expands upon the existing pre-operative, intra-operative, and post-operative knowledge and relationship between the anesthesiologists and the patient. Second it takes direct advantage of the well-established communication stream that currently flows between the surgeon and the anesthesiologist. Third it improves the quality and the efficiency of care by expanding the care team’s reach.

An individual surgeon’s ability to provide perioperative patient care is diminishing rapidly. Chronic non-communicable diseases like ischemic heart disease, cerebrovascular disease, and pulmonary disease are now so common as to be “the main causes of both disability and death worldwide” [75]. As noted above, not only is the overall prevalence of chronic disease increasing, the problem is also being made worse by lengthening lifespans. The introduction of universal healthcare, along with continued surgical advances, will fuel the demand for surgical services in the United States. Unfortunately, the resources required for delivering this care, including the number of trained surgeons are not increasing proportionally. Statistics from a June 2010 Association of American Medical Colleges (AAMC) report indicate that the growing shortage of practicing physicians, including surgeons, will reach over 90,000 by the year 2020 and will swell to over 130,000 by 2025 [76,77]. The future shortage of surgeons will be essentially equivalent to the shortage of primary care physicians [77]. The expansion of the perioperative care team—especially with anesthesiologists who communicate effectively with surgeons and possess an underlying familiarity with the patient and their medical and surgical history—will thus be critical to providing high *value* healthcare services in the future.

### Benefits to the specialty of anesthesiology

Anesthesiology as a specialty is in a unique position to improve surgical outcomes [3,78]. Standardized, evidence-informed perioperative care plans, designed in a multidisciplinary and cooperative team-based approach, will likely improve outcomes [13,79]. Anesthesiologists could be the common denominator for achieving this enhanced care for several reasons. Anesthesiologists have uniquely extensive training in preoperative evaluation, intraoperative management, postoperative and critical care, and pain medicine [80,81]. Anesthesiologists care for patients across the entire age range and spectrum of co-existing diseases. Anesthesiologists also typically manage complex operating and procedural schedules and lead perioperative care committees.

Like in many disciplines, physicians from other specialties and non-physician providers, who claim to deliver services with a similar quality, constantly encroach upon the traditional realm of anesthesiology [73,82]. This competition does little to improve care or reduce costs, but promotes piecemeal delivery with inherent quality risks [83]. In order to strengthen the future viability of the specialty, anesthesiology-based, coordinated care needs to offer a better service for health systems of the future [84]. To achieve this goal, it will be necessary to expand the core knowledge, skills, and experience expected of the perioperative anesthesiologist [3]. Anesthesiologists will need to view this movement toward becoming anesthesia perioperativist as an expansion of the specialty, rather than an abdication of their traditional intraoperative role [80,81]. The intraoperative role will remain strongly intact [82].

While not all anesthesiologists will be willing to play a role in this new activity, just as with the initial development of the anesthesia-based subspecialties of critical care medicine and pain medicine, a subset will need to do so and be supported by colleagues in their efforts. This expanded scope of anesthesiology may increase interest in the specialty among medical students and even internal medicine residents—the latter supported by the combined residency program recently created by the American Board of Anesthesiology and the American Board of Internal Medicine [85,86]. This notwithstanding, as promulgated by the ASA, the PSH will codify the role of the anesthesiologist as the primary perioperativist, making them more strategically vital and competitive [3,83].

### The healthcare economic advantages of the perioperative surgical home model

As noted earlier, in the new healthcare paradigm, all providers will be paid not just for the quantity of services provided, but rather how effectively and efficiently those services are delivered [37,38]. Instead of “No margin – No Mission,” the mantra first espoused by Sister Irene Krause of the Daughters of Charity National Health Care System, the new healthcare adage has become “No outcome – No income” [87]. To succeed in this new, heavily outcome-focused healthcare environment, physicians and their leadership need to rely far less on individual accomplishment but instead primarily on performance-based teams [88]. Our prototypic PSH at UAB is built upon a performance-based, integrated team of anesthesiologists and mid-level providers.

The medical community and the public are also increasingly embracing shared decision making, a process by which healthcare choices are made mutually by the clinician and the patient [6,7]. Shared decision making is viewed as the crux and the pinnacle patient-centered care [89,90]. In 1998 the IOM National Roundtable on Health Care Quality concluded that healthcare quality problems stem from the trio of “overuse, underuse, and misuse” of resources—a trio that remains paramount today [91,92].

Evidence shows that patient-centered care not only improves clinical outcomes, quality of life, and patient satisfaction [93,94] but also is associated with a decrease in inappropriate health care utilization in the primary care setting [95], especially in the setting of a patient-centered Medical Home [96,97]. A parallel positive effect on resources utilization can plausibly be expected with a patient-centered Perioperative Surgical Home.

Like all areas of healthcare, the specialty of anesthesiology is facing strong economic pressures that require a broader competitive strategy [83,98]. The austere economic landscape and the need for all types of healthcare systems, from academic medical centers to community hospitals, to provide a more coordinated and economical product dictate an urgent need for anesthesiologists to challenge our current successful business model and our assumptions about the market forces, mission, and core competencies of our specialty [83,84,98]. Value-based purchasing of health care [99-101], pay for performance [102,103], intense competition from other anesthesia care providers and proceduralists [82,98], and a changing payment paradigm that includes bundled payments or accountable care arrangements [104] are all powerful motivators to improve surgical care delivery and outcomes—particularly in the immediate perioperative setting. Maximizing the effectiveness, efficiency, and integration of delivered health care will not only reduce perioperative complications and improve healthcare [68,79,105], but is also good business practice [106].

Hospital-physician collaborations continue to evolve to include greater economic integration, including major financial gain and risk sharing [107,108]. Such greater financial integration can include an institutional funds flow model in which a negotiated work unit-based, relative-value system is implemented, with flexibility in allocation of funds (payments) [109]. Level of payment is based on the resources used by those care delivery teams achieving superior outcomes, thereby fostering innovation and reducing waste [110]. Such an integrated funds flow model and its complete quality and cost transparency are vital for future healthcare business success [111]. The successful PSH model will need to create strategic added value for a health system and payers [112]. This added value will strengthen the position of anesthesiologists as they navigate and negotiate in the face of finite, if not decreasing fiscal resources (i.e., making do with less).

## Summary

The Perioperative Surgical Home concept seeks to establish an anesthesiologist-led multidisciplinary system of coordinated and managed perioperative care throughout the entire surgical continuum. By implementing evidence-informed best practices, standardization of processes where applicable, patient-centeredness, and accountable management by a single coordinating service, patients are likely to get the most appropriate care possible. Eliminating overuse, underuse, and misuse of care, will likely lead to better outcomes at a lower cost—the definition of added value. This new system should also drive performance improvement and outcomes research to promote improved surgical care for all patients. Lastly, the expanded role played by anesthesiologists in the PSH should promote the continued vibrancy of the specialty.

## Abbreviations

CER: Comparative effectiveness research; PSH: Perioperative Surgical Home.

## Competing interests

All five authors declare that they have no financial, consultant, institutional or other relationships that resulted in bias or a conflict of interest in the conducting or reporting this study. The authors have no competing interests.

## Authors’ contributions

All five authors were involved in drafting the article and critically revising it for important intellectual content. All authors approved the final version to be submitted for publication. TRV was responsible for organizing the overall manuscript, and specifically for the Background, Summary, and the sections on holding the gains made in anesthesia-related patient safety and assimilating comparative effectiveness research. LAG was responsible for the section on achieving healthcare outcome metrics. AMB was responsible for the section on the benefits to the specialty of anesthesiology. TRH was responsible for the section on the perspective of the surgeon. KAJ was responsible for the section on its associated healthcare economic advantages. JFP was responsible for the section on a comparison with the hospitalist model of perioperative care.

## Authors’ information

TRV is the Maurice S. Albin Professor and Vice Chair of Pain Medicine in the Department of Anesthesiology at the University of Alabama at Birmingham. He holds an MPH in Clinical Outcomes.

LAG is an anesthesiology resident in the Department of Anesthesiology at the University of Alabama at Birmingham. He holds an MPH in Epidemiology.

AMB is a Professor and the Vice Chair for Quality and Patient Safety in the Department of Anesthesiology at the University of Alabama at Birmingham and the Chief of Staff of the UAB Health System.

TRH is a Professor of Surgery and the J.D. Sherrill Chair of Orthopaedics at the University of Alabama at Birmingham.

KAJ is the Alfred Habeeb Professor and Chair of the Department of Anesthesiology at the University of Alabama at Birmingham.

JFP is a Professor and the Vice Chair of Critical Care and Perioperative Medicine in the Department of Anesthesiology at the University of Alabama at Birmingham.

## Pre-publication history

The pre-publication history for this paper can be accessed here:

http://www.biomedcentral.com/1471-2253/13/6/prepub
